# Impact of newborn screening for cystic fibrosis on clinical outcomes of pediatric patients: 10 years’ experience in Lodz Voivodship

**DOI:** 10.1186/s13052-021-01040-5

**Published:** 2021-04-09

**Authors:** M. Olszowiec-Chlebna, E. Mospinek, J. Jerzynska

**Affiliations:** Department of Pediatrics and Allergy, Medical University of Lodz, Copernicus Memorial Hospital, Korczak Paediatric Center, Piłsudskiego 71 Str, 90-329 Lodz, Poland

**Keywords:** Cystic fibrosis, New born screening, Diagnosis, IRT/DNA protocol, Children

## Abstract

**Background:**

Cystic Fibrosis newborn screening (CFNBS) is the optimal method to diagnose the disease during the asymptomatic period. The aim of the study was to determine how CFNBS affects long term clinical outcomes.

**Methods:**

Data from infants who were born in Lodz Voivodship, referred to CF center as a part of CFNBS according to IRT/DNA protocol were compared to the data of children with established CF diagnosis before the start of NBS in Poland (Group CF, *n* = 52).

**Results:**

In 37 children (during 151 referred infants) the diagnosis of CF was established due to CF NBS (CF NBS Group, *n* = 37). The average time of diagnosis was 1.59 month in Group CF NBS and 45.25 months in 52 children from Group CF.

Pulmonary exacerbations occurred on average 4.2 times in Group CFNBS and they were hospitalized on average 0.5 times compared to Group CF – respectively 6.77 and 2.14 (*p* < 0.001).

The number of PA infected patients increased between the fifth and eighth year of age (OR = 1.16 (95% CI: 1.04–19) (*P* = 0.007)) regardless of the study group (*P* = 0.984). Patients with MRSA infection have a higher risk of PA infections in subsequent years of their life (OR = 1.45 (95% CI: 1.03–2.03) (*P* = 0.032)).

**Conclusions:**

CF NBS has beneficial effects primarily on decrease of pulmonary withhope for a longer life expectancy and better and centralised treatment in multidisciplinary CF focused centres.

## Introduction

Cystic fibrosis (CF) is the most common fatal autosomal recessive disease in Caucasians [1]. It is widely recognized that CF is a variable condition, that may affect the respiratory tract, pancreas, intestine, male genital tract, hepatobiliary system and exocrine sweat glands, resulting in complex multisystem disease. The severity of clinical symptoms results due to pathogenic mutations in both alleles of the CF transmembrane conductance regulator (CFTR) gene. More than 1950 mutations have been described so far in the CFTR gene, grouped in 5 classes based on the impact on protein synthesis or activity [2, 3].

Over the last 50 years, European countries have introduced newborn bloodspot screening (NBS) for a range of inherited diseases as an important public health program. In 2004, the European CF Society established the Neonatal Screening Working Group (NSWG) to support the implementation of CF NBS program across Europe [4, 5]. CF NBS is the optimal method to diagnose the disease during the asymptomatic period. The primary goal of newborn screening is to decrease morbidity, mortality and associated disabilities. The early diagnosis is associated with improved physical development and slower lung deterioration due to the lower frequency of infections.

All CF NBS programs begin with detection of an elevated immunoreactive trypsinogen (IRT) level in a dried blood specimen from the newborn [3, 5, 6]. A positive IRTscreen is triaged to second-tier testing, which is DNA mutation testing in many national screening programs, including Poland (IRT/DNA protocol) [3–7]. Despite the advent of NBS and improved knowledge about CFTR genetics, CF diagnosis remains incomplete for many reasons, such as inconclusive sweat chloride values, CFTR mutations of uncertain pathogenicity, and differential expression of CFTR or modifier effects [8]. Also CF NBS introduced a new complexity and diagnostic dilemma, namely infants with abnormal screening tests, because of elevated immunoreactive IRT levels but inconclusive sweat tests and/or DNA results. The term for infants with an inconclusive diagnosis have been proposed: CF screen positive, inconclusive diagnosis (CFSPID) in Europe [9].

The first pilot NBS CF study was performed in Poland from 1999 to 2002 and included about 25% of the Polish population. The current program was implemented in 2006 and was expanded to the whole country in the summer of 2009. In Lodz Voivodeship, CF NBS was introduced on 1st June 2009 [3, 6, 7].

The research presents 10 years of experience of our CF center with the national newborn CF screening program in Lodz Voivodship and including the analyzis of diagnostic dilemma occurred during the program. The aim of the study was to determine how early diagnosis of CF, using neonatal screening with IRT/DNA protocol, affects long term clinical outcomes in comparison to CF patients with disease diagnosed before CF NBS?

## Material and methods

### Patients

#### Patients participated in the screening program

The study population comprised of infants who were born between 01.07.2009 and 31.12.2019 in Lodz Voivodship and were referred to our CF center as a part of NBS for CF and performed CFTR gene analysis according to IRT/DNA protocol described below. The infants had been evaluated at least once per 3 months. They were divided to three groups according to laboratory tests and further clinical evaluation as CF NBS Group, CF SPID and false positive NBS. Cystic fibrosis **(CF NBS Group)** was diagnosed in infants based on the level of sweat chloride in addition to evidence of CFTR dysfunction. The designation **CF SPID** was established to asymptomatic infants if they presented a positive CF NBS test plus: sweat chloride < 59 mmol/L and 2 CFTR mutations with 0–1 CF-causing CFTR mutations. The asymptomatic CF NBS positive infant with presence of no CFTR mutation or mutation in one allele plus a negative sweat test, referred as **false positive NBS** (including carriers).

#### Patients diagnosed with CF before introduction of screening program

We also analyzed data of 52 patients (24 females and 28 males) with established CF diagnosis born between 1996 and 2009 (before the newborn screening in Poland) attending our Cystic Fibrosis Outpatient Center in Copernicus Hospital in Lodz, Poland. Patients in this group had been referred to the Center due to occurrence of symptoms suggesting CF **(CF Group).**

Next, we compared both groups CF NBS and CF. We have chosen the interval - between 5 and 8 years of age to compare both groups, because at that time the most data were available.

### Data assessment

In both groups the data were obtained retrospectively after a review of patient charts. The socio-economic data were analysed. These included a place of living defined as a large city above 100,000 inhabitants, a small city 10,000–100,000 inhabitants, and a village below 10,000 inhabitants. The perinatal period contained data: delivery data and weight, Apgar score and presence of meconium ileus (MI), time of CF diagnosis. We also analysed body weight and percentile of body weight at 5 and 8 years of age.

### Clinical evaluation

Respiratory status was assessed by the forced expiratory volume in 1 s (FEV1) in spirometry, expressed as percentages of predicted values for height, weight, age, and gender. All analyzed measurements were done in fifth and 8 year of life, only in stable periods (using a Jaeger MasterScreen Body spirometer; E Jaeger GmbH; Wurzburg, Germany). The tests were performed according to American Thoracic Society standards [10].

The number of bronchopulmonary exacerbations and number of hospitalizations (due to pulmonary disease, longer than 3 days) between five and 8 years of life were evaluated. Pulmonary exacerbation was defined as excessive sputum expectoration, general malaise, and need for antibiotics. Chronic bacteria colonization *Pseudomonas aeruginosa* (PA) or methicillin-resistant *Staphylococcus aureus* (MRSA) were also collected, and defined as three positive consecutive sputum cultures over a period of 6 months in fifth and 8 year of life.

### CF NBS protocol

IRT was measured in dried blood samples from 3 to 5 days old neonates. The IRT concentration cut-off was established as > 99.4 percentile (according to the pilot CF NBS program [6]). The elevated IRT caused further genetic testing from the same dried blood samples. In neonates with meconium ileus (MI), the DNA analysis was performed regardless of IRT level. The measurements of IRT (by using IRT Neonatal Screening ELISA colorimetric assay - IBL International) and DNA analysis from sampling paper (processing with the Extract Blood PCR Kit) were performed in the Genetic Department, Institute of Mother and Child, Warsaw, Poland. Since September 2011, the extended DNA analysis panel has been used and comprised 95% of mutated alleles in the Polish population.

Neonates with one or two mutations in CFTR detected due to NBS examinations were directed to CF Centres for clinical assessment and sweat tests (measured by the quantitative pilocarpine iontophoresis method and Nanoduct method parallel) [6]. CF NBS was conducted as a pilot programme in four Polish districts in the period 1999–2003. In 2006 CF NBS started again in the same regions and was gradually extended across the country. In June 2009 covered all of newborn population in Poland. In the 1980s, the prevalence of CF for Caucasians was estimated at 1: 2500 live births. The disease incidence was more accurately calculated to 1: 4000–5000 thanks to the extensive implementation of CF NBS in the world. In Poland due to data from CF NBS in the years 2006–2010 it is believed that one child every 4394 live births is born with CF.

The study was approved by the Ethical Committee of the Medical University of Lodz, Poland (nr RNN/145/20/KE).

### Statistical analysis

Numerical traits were described by way of the arithmetic mean, standard deviation (SD), standard error (SE), when applicable, and their minimum-to-maximum values. Categorical variables were depicted by using counts (n) and percentages (%).

Multiple logistic (for binary dependent variables), multiple linear (for numerical traits), and multiple Poisson (for count data) regression models were fitted in order to test statistical co-dependencies. When dealing with non-normally distributed variables, robust standard errors (i.e. sandwich estimators) were used in the regression equations. All the regression models were controlled for the studied patients’ characteristics such as sex, gender, place of residence, birth weight, APGAR and also for crucial infections (PA, MRSA) before their 5th year of age. Missing data were case-wise deleted.

A level of *P* < 0.05 was deemed statistically significant. All the computations were performed by using of Stata/Special Edition, release 14.2 (StataCorp LP, College Station, Texas, USA).

## Results

### Evaluation of patients participated in the screening program (CF NBS positive infants)

One hundred fifty one infants were referred to our CF center as a part of NBS for CF due to CF suspicion: 84 females and 67 males. The CFTR gene analysis for each positive IRT, and neonates with MI regardless of IRT levels, allowed to identify mutations in one allele in 145 patients and in both alleles in 44 patients (96.03% of screened CF patients). Among 44 cases, after biochemical, genetic and clinical evaluations, in 37 children the diagnosis of CF was established (24.5%) during the first verifying visit (**CF NBS Group**) - in 76% the diagnosis was establish up to 35 days after birth, in 9 patients the first verifying visit took place later in life due to hospitalization connected with their MI. The mean birth weight in CF NBS patients was 3226.34 g, In this group, 9 patients had MI (24.32%). In the remaining 7 (out of 44) cases (15.9%), despite detection of two CFTR mutations, a clinical evaluation did not confirm the CF diagnosis (**CF SPID)**. These infants had the CFTR genotype as follows: 3 patients with [F508del]; [IVS8-5 T+(TG)11], [3849 + 10kbC > T];[R117H], [Y301C];[3271 + 18C > T], [V754M];[V562L], [F508del];[R117H]. The above mutations found at least in one allele causing unproven or uncertain clinical consequences. These patients remain under the observation of our clinic. The most common mutation was F508 del, detected in 104 patients in one or both alleles (68.88%).

Six patients (3.97%) who were directed to the Cystic Fibrosis Outpatient based on the IRT elevations, had DNA analysis for CF mutations negative and they sweat chloride test was correct (**false positive NBS**). After further observations and examinations, the CF was excluded. So far (until December 2019) we also have information about two cases of false-negative results in CF NBS in our region (**false negative NBS)**.

### Comparison of group CF NBS and group CF

At the time of data collection 27 of CF NBS patients were at least 8 years old, remain under the medical care in our CF Outpatient Center and were included into compared analysis. The Group CF NBS with positive result of newborn CF screening included: 59% females, mean birth weight 3168.52 g and mean Apgar 8.78. Mutation F508 del was detected in both alleles in 29.63% patients.

Fifty two patients belonging to the **Group CF,** were born between 1996 and 2009 (before the start of newborn screening in Poland) – 24 females and 28 males. They were included into compared analysis with Group CF NBS**.** Their mean birth weight was 3195.6 g, Apgar 8.54, F508 del mutation in both alleles was detected in 50% cases.

Group CF NBS and Group CF demographic and clinical characteristics are shown in Table 1.

**Table 1:** Descriptive characteristics CF patients by screening status

***Investigated trait***	***Statistical parameters***	
	Screened Group CF NBS (***n*** = 27)	Unscreened Group CF (***n*** = 52)
**Sex, n (%)**		
**Females**	16 (59.26)	24 (46.15)
**Males**	11 (40.74)	28 (53.85)
**Birth weight (g),**		
**mean (SD), min.-max.**	3168.52 (715.10), 970–4220	3195.60 (526.03), 2330–4400
**median (Q**_**1**_**-Q**_**3**_**), min.-max.**	3310 (2800–3620), 970–4400	3190 (2800–3600), 2330–4400
**APGAR rating,**		
**mean (SD), min.-max.**	8.78 (1.69), 3–10	8.54 (1.88), 1–10
**median (Q**_**1**_**-Q**_**3**_**), min.-max.**	9 (8–10), 3–10	9 (9–9), 1–10
**F508del mutation, n (%)**		
**overall**	24 (88.89)	46 (88.46)
**in both alleles**	8 (29.63)	26 (50.00)
**Meconium ileus, n (%)**	9 (33.33)	9 (17.31)
**Concentration of chlorides (mmol/l),**		
**mean (SD), min.-max.**	98.93 (25.14), 10–123	100.83 (20.36), 60–152
**median (Q**_**1**_**-Q**_**3**_**), min.-max.**	106 (89.117), 10–123	100 (90–110), 60–152

(*Abbreviations:* *n* number; *SD* standard deviation; *Q*_*1*_ lower quartile; *Q*_*3*_ upper quartile; *min* minimum value; *max.* maximum value)

The average time of the CF diagnosis was 45.25 months from birth in **Group CF.**

and 1.59 months in **Group CF NBS**.

The most significant difference between this two Groups was the number of exacerbations and hospitalizations between 5 and 8 years of life (*p* < 0.001). Pulmonary exacerbation in analysed period occurred on average 4.2 times in children in **Group CF NBS** and they were hospitalized on average 0.5 times. While in **Group CF** children mean of pulmonary exacerbations was 6.77 and mean number of hospitalization was 2.14.

Even though the average body weight and its percentile value was higher in the Group CF NBS, this difference was not significant. What is more weight gain between 5 and 8 years old was greater in the Group CF. No statistically significant predictors affecting weight gain in both groups were defined.

In spirometry measurements patients in both groups presented stable values of FEV1 during at least 3 years of observation, and the difference was not significant.

The number of PA infected patients increased significantly between the fifth and eighth year of age of the examined children (OR = 1.16 (95% CI: 1.04–19) (*P* = 0.007)) regardless of the study group (*P* = 0.984). It seems that patients with MRSA infection in the fifth year of life have a higher risk of PA infections in subsequent years of their life (OR = 1.45 (95% CI: 1.03–2.03) (*P* = 0.032)). The number of patients with PA or MRSA infection in the fifth year of life and PA infection in the age of 8 was greater in the CF Group, but these differences were not statistically significant.

Descriptive characteristics of all CF patients by screening status are shown in Table 2.

**Table 2:** Descriptive characteristics of patients in Groups CF NBS and Group CF

***Investigated trait***	***Statistical parameters***		
	Group CF NBS (***n*** = 27)	Group CF (***n*** = 52)	***P***-values
**Body Weight in the 5th year of life (kg),**			0.201
**mean (SD), min.-max**	18.30 (1.92), 15.00–21.00	17.77 (2.43), 14.50–23.00	
**median (Q**_**1**_**-Q**_**3**_**), min.-max.**	19 (17–20), 15.00–21.00	17 (16–20), 14.50–23.00	
**Body Weight in the 8th year of life (kg),**			0.312
**mean (SD), min.-max**	25.27 (1.85), 22.00–28.00	25.11 (4.60), 19.00–38.00	
**median (Q**_**1**_**-Q**_**3**_**), min.-max.**	25 (24–27), 22.00–28.00	24.50 (21–27), 19.00–38.00	
**Absolute weight gain 5–8 years (kg),**			0.777
**mean (SE), min.-max.**	6.57 (0.46), 3.00–11.50	7.23 (0.48), 2.50–15.00	
**median (Q**_**1**_**-Q**_**3**_**), min.-max.**	6.50 (5–8), 3.00–11,.50	6.00 (5–8), 2.50–15.00	
**Date of CF diagnosis (month of life),**			**< 0.001**
**mean (SD), min.-max.**	1.59 (0.9), 2.7	45.25 (47.77), 1–180	
**median (Q**_**1**_**-Q**_**3**_**), min.-max.**	2 (2–3), 2–7	34.50 (6–66), 1–180	
**Infection of** ***PA*** **in the 5th year, n (%)**	7 (26.92)	17 (40.48)	0.256
**Infection of** ***PA*** **in the 8th year, n (%)**	10 (50.00)	27 (58.70)	0.513
**Infection of** ***MRSA*** **in the 5th year, n (%)**	6 (22.22)	13 (30.23)	0.463
**Number of exacerbation in 5–8 years,**			**< 0.001**
**mean (SD), min.-max.**	4.20 (2.50), 2–9	6.77 (3.18), 2–14	
**median (Q**_**1**_**-Q**_**3**_**), min.-max.**	3 (2–5), 2–9	6 (4–9), 2–14	
**Number of hospitalizations in 5–8 years,**			**< 0.001**
**mean (SD), min.-max.**	0.50 (0.61), 0–2	2.14 (1.82), 0–9	
**median (Q**_**1**_**-Q**_**3**_**), min.-max.**	0 (0–1), 0–2	2 (1–3), 0–9	
**FEV1 in the 5th year (% of pred. value),**			0.721
**mean (SD), min.-max.**	91.93 (10.22), 72–105	89.75 (17.12), 47–119	
**median (Q**_**1**_**-Q**_**3**_**), min.-max.**	97 (82–99), 72–105	88 (81–105), 47–119	
**FEV1 in the 8th year (% of pred. value),**			0.308
**mean (SD), min.-max.**	92.30 (13.49), 65–110	88.20 (20.64), 42–138	
**median (Q**_**1**_**-Q**_**3**_**), min.-max.**	94 (83–104), 65–10	89 (77–98), 42–138	

(*Abbreviations:* *n* number; *SD* standard deviation; *Q*_*1*_ lower quartile; *Q*_*3*_ upper quartile; *min.* minimum value; *max.* maximum value)

## Discussion

According to the Neonatal Screening Working Group new-born screening for CF provides an immediate diagnosis, before the onset of clinical symptoms [11–13]. In the beginning CF NBS raised doubts about ethical aspects with regard to possible benefits and risks. After many years of experience CF NBS has been widely implemented and accepted. Ten years of CF NBS programme in Lodz Voivodship leads to diagnosis of CF in 37 neonates among 151 children directed after their birth with CF suspicion to our Outpatient Center. The incidence of CF in Poland based on neonatal screening is currently 1:4394–1:5000 [3, 6].

CF NBS has been based on the assumption that pre-symptomatic detection permits early access to specialised medical care, and thus results in less morbidity and longer life expectancy. In our region CF NBS screened patients were diagnosed and treated in multidisciplinary CF centre between first and second month of their life. For comparison the median age of the diagnosis of patients, who were born before the start of CF NBS in Poland due to typical CF symptoms – 45.25 months from their birth. According to the literature, the most benefits associated with early identification of CF including include better growth and lung function, less intensive therapeutic burden and reduced cost of care [11–14]. Our observations show that early diagnosis and the introduction of appropriate treatment for asymptomatic CF patients resulted in a lower frequency of the pulmonary exacerbation and lower number of hospitalizations. It is very beneficial observation due to reports which emphasise that the repeated mild-to-moderate pulmonary exacerbations, especially in the first years of life result in airway remodelling. While more severe hospitalised episodes could increase structural airway injury risk [13–16]. Data from Australia showed improved survival in future in patients with lower number of hospitalisations in the first 3 years of life [17]. The occurrence of respiratory exacerbations are associated with an accelerated decline in lung function and reduced quality of life and survival [15–17]. On the other hand mean values of FEV1 in the analysed patients, presented stable results during at least 3 years of observation; there was no difference between Groups. This may be due to the age - below 8 - too early to identify significant differences in FEV1 secondary to clinical outcome (such as pulmonary exacerbations, bacterial colonisations), which likely takes longer to cause impairment of lung function that is identifiable with spirometry [16].

Children with CF are highly susceptible to chronic respiratory tract colonization and subsequent recurrent infection. It is know that once chronic PA infection is established, the risk of mortality and morbidity increases [18, 19]. In our study the mean number of patients with PA or MRSA colonization in the 5 years old and PA chronic infection in the age of 8 was lower among children screened in NBS, but there were no significant differences between both groups. The number of PA infected persons increased significantly between the fifth and eighth year of age regardless of belonging to the study group. Although the increasing number of SA and PA colonized patients were observed with age in both CF NBS and CF group, the CF NBS children had fewer exacerbations and hospitalisations due to intensively treatment from the very first PA sputum detection. The important observation was that patients in both groups with SA infection in the fifth year of life have a higher risk of PA infections in subsequent years of their life. Persistent presence of MRSA in the respiratory tract is associated with increased rates of PA through mediation of PA biofilm formation [18, 19]. In practice, we suggest to employ intermittent, symptom-based treatment of SA especially MRSA to delay initial PA colonization. The colonization with PA remains the most ordinary airway pathogen contributing to shortened survival in CF patients. For over a two decades antibiotic effective for PA (for example tobramycin inhalation solution (TIS)) is recommended for the treatment and early introduction with TIS has resulted in improved lung function and reduced risk of airway exacerbations [20, 21]. In our study in CF group the diagnosis was established mean in 42.25 month of life, but even maximally in 180 months of life. In such situation at the time of CF recognition patients’ sputum were often positive for PA and we treated PA colonization not an initial infection. Patients from CF NBS group were treated intensively from the very first PA sputum detection. We believe this may have contributed to a reduction in the number of pulmonary exacerbations and associated hospitalizations. Moss et al. [21] proved that delay in initiation of TIS therapy is associated with reduced long-term improvement over baseline, suggesting an irreversible component to lung function decline. To be maximally effective, TIS treatment should begin early after first detection of PA. In agreement to this study, although the incidence of PA found in the airways became similar over the years in both study groups (CF and CF NBS) the number of exacerbations and hospitalizations in CF NBS group was significant lower.

CF NBS allows the affected infants to receive immediate treatments including not only physiotherapy and preventing of pulmonary manifestations of the disease, but also great emphasis is pleased on assessment of nutritional status, and pancreatic enzyme replacement therapy. The Wisconsin randomised trial on newborn screening for CF showed that screened neonates exhibit better nutritional status in the first years of life [22], but no output is available from this cohort on long-term survival. We observed trend that body weight were higher in the CF NBS Group and they presented systematic and stable weight gain but this results were not statistically significant.

According to data published by Bobadilla et al., the complete CFTR gene analysis by sequencing of selected regions should identify mutations in one or both alleles in nearly 95% of screened children [23] and IRT/DNA strategy to achieve sensitivity close to 100% (despite children with MI, whose IRT level is not elevated). Due to Fritz formula the predicted false-negative rate for the Polish population is 6–7 cases per year, which gives 1–2 omitted CF cases per 100,000 live births [24]. It shows that despite the high sensitivity and specificity of IRT/DNA protocol it is important to follow patients with CF suggestive symptoms even when CF NBS was negative. We had two cases of false-negative results in CF NBS in our region to this day. According to normal values of the IRT in new-borns with MI they are in risk group of a false-negative first step of CF NBS protocol. Each case of MI should be reported to the NBS center by appropriate note on sampling paper and neonates must to be verify in CF centers. In our study, the new-borns with MI represent 24% of CF diagnosis, while the literature data shows MI frequency about 10–20% [25].

The IRT/DNA strategy allows to detect not only CF disease but also carries of the CFTR gene mutations, as well as cases of unclear clinical consequences. This fact constitutes a definite psychological problem for patients and their parents and raised doubts about ethical aspects of the CF NBS. It may have negative influences on further procreation of such persons. We detected 101 patients with mutations only in one allele of CFTR gene (what was 66.89% of all neonates from CF NBS). They were defined as carriers and in all cases, a genetic consultations for their parents and siblings was recommended.

Some limitations of our current study include a single centre analysis, which limits its generalizability. What is more we made the comparison to historical data (a blinded controlled study with CF- newborns left untreated was unacceptable due to ethical reasons).

Over the years new therapies were approved, standards of care established therefore, the outcomes of same patients in subsequent years is expected to be improved, even without the advent of newborn screening. We have to choose the appropriate time interval to compere a historical cohort (CF Group) and CF NBS positive patients.

## Conclusion

In conclusion, newborn screening contributes to early diagnosis of cystic fibrosis. The presented study showed that CF NBS has beneficial effects primarily on decrease of pulmonary exacerbations with hope for a longer life expectancy in these group.

## Data Availability

The datasets used and/or analysed during the current study are available from the corresponding author on reasonable request.

## Abbreviations

CF: Cystic fibrosis
CFNBS: Cystic Fibrosis newborn screening
CFTR: CF transmembrane conductance regulator
FEV1: The forced expiratory volume in 1 s
IRT: Immunoreactive trypsinogen
MI: Meconium ileus
NSWG: Neonatal Screening Working Group
PA: *Pseudomonas aeruginosa*
MRSA: Methicillin-resistant *Staphylococcus aureus*
TIS: Tobramycin inhalation solution
